# Optimization of Internal Chip Evacuation Cutting Tool System for Deep Bottle Holes Based on Chip Morphology Control

**DOI:** 10.3390/ma18184263

**Published:** 2025-09-11

**Authors:** Yazhou Feng, Zixiang Xu, Wanzhong Li, Kaining Shi, Yang Zhang, Weiye Yang

**Affiliations:** 1 Mechanical Engineering College, Xi’an Shiyou University, Xi’an 710065, China; asian5921@126.com (Y.F.); liwanzhong@xsyu.edu.cn (W.L.); zhangy20011201@163.com (Y.Z.); m13785760675@163.com (W.Y.); 2 School of Mechanical Engineering, Northwestern Polytechnical University, Xi’an 710072, China; shikaining@nwpu.edu.cn

**Keywords:** deep bottle hole, deep hole boring, deep hole tool, chip morphology, cutting parameter

## Abstract

Complex deep hole parts are crucial for major equipment to achieve structural innovation and technological leapfrogging. With the continuous advancement of requirements for weight reduction, efficiency enhancement, and performance modification, the application of deep bottle hole parts has become increasingly widespread. Their structures are mainly characterized by complex interior profiles, variable diameters, large depth-to-diameter ratios, etc. However, the traditional vibration-damping tool boring process is prone to problems such as poor hole straightness and low cutting efficiency due to poor tool rigidity and difficult chip evacuation. For this reason, this research focuses on an internal chip evacuation tool system for deep bottle holes based on cutting morphology control. First, based on the structural characteristics of deep bottle hole components, a specialized tooling system with three guide pad supports and internal chip evacuation channels was designed. Subsequently, the tool’s chip evacuation channel was optimized using fluid simulation results from the tooling system, and the coupled relationship between chip morphology and chip evacuation efficiency was analyzed. Finally, a segmented and layered boring process scheme was proposed based on the component’s structural features. Through deep bottle hole-boring experiments, the surface roughness of the hole interior reached 0.9 µm, and eccentricity was reduced by 54.39%, confirming that the scheme effectively forms chip morphology into spiral curled chips and validating the feasibility and effectiveness of the tooling system.

## 1. Introduction

In the field of mechanical machining, deep hole drilling is a key technical branch. Currently, machining is no longer limited to the basic definition of a length-to-diameter ratio (L/D) greater than 10, and there is a demand for many complex types of deep hole parts [1,2]. Among them, deep bottle holes are the most typical, with structural characteristics of being small at both ends and large in the middle, like a bottle shape [3,4]. Such holes can reduce the weight of key components without affecting surface integrity and improve performance [5]. Their machining usually adopts the boring method. The boring method for deep bottle holes is called bottle boring, internal profiling, or chamber boring, which is a special cutting method. This method creates blind holes, and when boring their inner surfaces, the tool must change the radial angle while ensuring rigidity to achieve complex machining of non-cylindrical inner surfaces [6]. Bottle boring for deep bottle holes is mostly applied to through-hole machining. In this case, the tool does not need to be designed with internal chip evacuation channels, and chips will be directly discharged from the inside of the workpiece, driven by high-pressure cutting fluid. However, for deep blind hole parts, the machining process is semi-closed, making it difficult for cutting heat to dissipate. It is necessary to use coolant to cool the tool [7,8,9], and to avoid chips damaging the machined surface, it is required to design chip evacuation channels inside the tool [10]. However, the structural design of chip evacuation channels will directly affect issues such as chip evacuation, thereby affecting machining accuracy [11].

Therefore, the design of the tool structure is of great importance. Inadequate chip evacuation will not only lead to problems such as chip blocking, increased torque, and tool breakage, but could even result in the scrapping of machined parts [12,13]. Thus, the ability to achieve good chip breaking and smooth chip evacuation directly affects the machining quality of deep blind hole parts [14,15]. It is necessary to study specific issues such as tool structure design, chip evacuation, and cutting fluid flow to ensure high chip evacuation efficiency during machining and prevent chip accumulation. Due to the above problems, the machining of deep bottle holes has become a major difficulty.

Researchers have conducted numerous theoretical and experimental studies on the boring of deep blind bottle holes. Fritsching et al. [16] applied visual analysis technology to typical machining processes. Through shadowgraph imaging and flow measurement, they explored the flow and working states of cutting fluid in different machining processes. Based on the analysis of these states, efficient machining parameters and the application range of cutting fluid dosage can be derived. Schnabel et al. [13] addressed problems such as large chip volume and difficult evacuation in the machining process of the Boring and Trepanning Association (BTA) system, and studied and adopted a topological optimization strategy to expand the cross-sectional area of the chip evacuation port, which significantly improved chip evacuation performance. Gerken et al. [17] designed a new type of deep bottle hole machining system, which can perform contour machining in axial and radial directions, and studied and discussed the future improvement of the system from fully mechanical drive to electric drive. Wu et al. [5] designed a special boring tool for deep bottle holes. Based on the Single-Tube Internal Evacuation Drill, the motor horizontally drives the wedge plate movement mechanism inside the tool bar to convert the radial movement of the tool into axial movement, thereby machining bottle-shaped holes in straight holes. Metzger et al. [18] designed a tool capable of machining variable-diameter holes inside deep holes. Its diameter variation mechanism is mainly based on the inclined plane transmission principle, and the tool is equipped with internal chip evacuation channels. Although it has high machining accuracy, the chip evacuation effect is not smooth and the machining efficiency is low. Oezkaya et al. [19] used Computational Fluid Dynamics (CFD) to study the cutting fluid flow of the above-mentioned tool, simulated the distribution of cutting fluid during the machining process, and found that the cutting fluid power was seriously insufficient during machining, and thus, it could not effectively carry chips out and was very likely to cause chip blocking. Oezkaya et al. [20] used Computational Fluid Dynamics–Discrete Element Method (CFD-DEM) coupling technology to simulate the movement of chips and cutting fluid. By improving the tool structure, the flow velocities of cutting fluid and chips increased by 40% and 60%, respectively. Oezkaya et al. [19] used CFD to simulate the distribution of cutting fluid during deep hole machining. The study showed that when the cutting fluid carries chips to the chip evacuation inlet, it is difficult to introduce chips with large-radius curls into the channel, thus concluding that the smoothness of chip evacuation is closely related to the morphology of chips.

Current research primarily focuses on the chip morphology and chip evacuation characteristics of deep bottle holes under complex machining conditions. However, studies on the boring process of deep blind bottle holes are relatively limited. Existing boring tools fail to ensure efficient chip evacuation for such deep holes, and there is a lack of analysis on the correlation between machining parameters and chip shape. Additionally, discussions regarding the final machining accuracy of the finished product are absent. Therefore, it is necessary to study the tool system and chip morphology according to the structural characteristics of deep bottle hole parts. A special deep hole tool device is designed to improve the rigidity of the machining system, and based on the fluid–solid coupling principle, the cutting parameters are optimized to realize chip control and reduce the deviation of the hole axis.

## 2. Deep Blind Hole Tool System Design

### 2.1. Tool Device and Machining System

**Figure 1:**
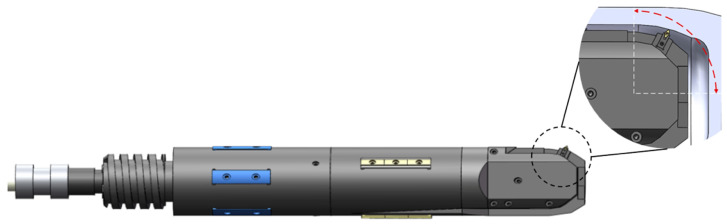
Deep blind hole-boring tool.

**Figure 2:**
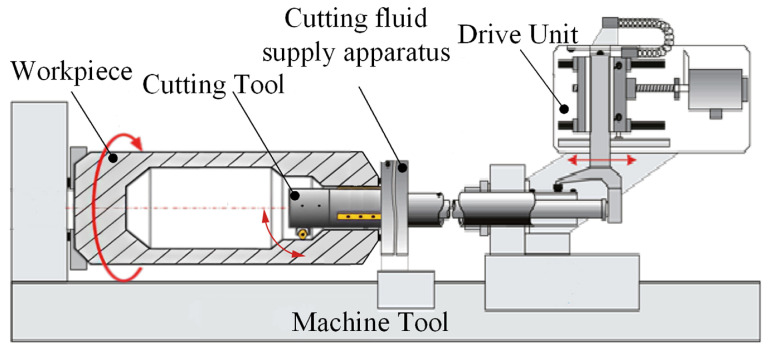
Deep bottle hole-boring system.

For deep blind holes of bottle structure parts with a length-to-diameter ratio greater than 15 (L/D ≥ 15), a boring tool enabling radial retraction within the bore was engineered, with a chip evacuation channel integrated into the tool’s interior. This tool is driven by an external drive unit, which causes the internal pull rod to undergo axial extension and retraction. Through a gear-and-rack mechanism, axial motion is converted to rotational movement of the cutting bit, achieving radial angular adjustment to facilitate multi-axis linkage cutting during deep bottle hole machining, as shown in Figure 1. Additionally, a dedicated machining system was developed for deep blind holes of bottle structure parts. This system comprises the boring tool, cutting fluid supply apparatus, drive unit, and rail, as shown in Figure 2.

### 2.2. Guide Pad Distribution Model

**Figure 3:**
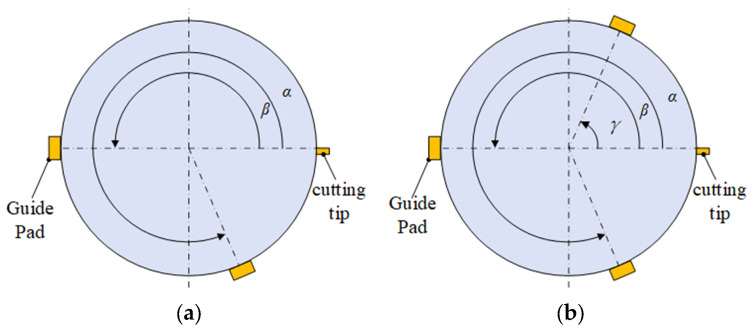
Guide pad structure. (**a**) Two-guide-pad structure. (**b**) Three-guide-pad structure.

Deep hole machining tools often use a two-guide-pad structure. To improve the stiffness of the deep blind hole-boring tool system, an additional guide pad was added to the original tool structure through computational analysis, thereby enhancing the overall system stiffness. In the deep hole-boring process, the three-guide-pad structure, due to the presence of a third guide pad providing auxiliary support, can balance out the counteracting forces of the other two guide pads [21]. Compared to the two-guide-pad structure, as shown in Figure 3a. The three-guide-pad structure demonstrates better performance. Therefore, the tooling design adopts the three-guide-pad structure for guide pad arrangement, as shown in Figure 3b.

**Figure 4:**
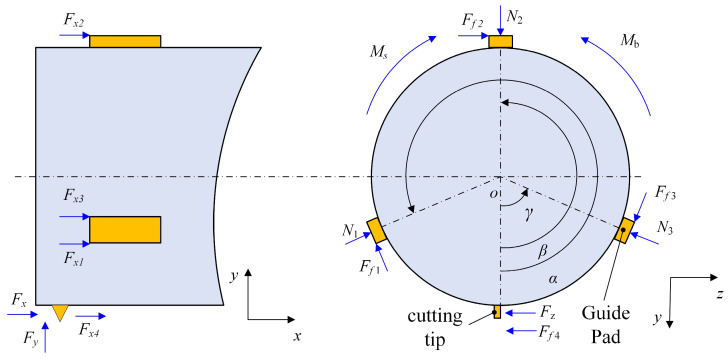
Tool force diagram during machining process.

Figure 4 shows the force distribution on the tool during the deep hole-boring process. The cutting tip primarily experiences axial force $F_{x}$, radial force $F_{y}$, and tangential force $F_{z}$. Additionally, there is a friction force $F_{f4}$ between the tool tip and the inner surface of the deep hole. Furthermore, friction force $F_{fx4}$ is present between the secondary cutting edge and the inner surface of the deep hole. The guide pads maintain interference fit with the deep hole inner surface, generating friction forces $F_{f1},F_{f2},F_{f3}$, axial friction components $F_{fx1},F_{fx2},F_{fx3}$, and extrusion forces $N_{1},N_{2},N_{3}$, during workpiece rotation, where $M_{s}$ is the resultant moment of the cutting force $F_{zi}$ at point O; $\;M_{b}$ is the support moment from the tool bar to cutting head.

As derived from the aforementioned mechanical model, the resultant cutting force and resultant moment are expressed as

(1) $$\left\{\begin{array}{l} F_{hor}=\sum F_{yi}\\ F_{ver}=\sum F_{zi}\\ M_{s}=\sum m_{O}F_{zi} \end{array}\right.$$

where $F_{hor}$ is the resultant cutting force in the horizontal direction; $F_{ver}$ is the resultant cutting force in the vertical direction; $F_{yi}$ is the tangential force in the *y*-direction; $F_{zi}$ is the tangential force in the *z*-direction; $M_{s}$ is the cutting force moment at point O.

Based on the force equilibrium during the machining process, force F is considered negligible, the force conditions of the deep blind hole-boring tool on the y-z plane can be obtained.

(2) $$F_{hor}+N_{1}\cos\alpha +N_{2}\cos\beta +N_{3}\cos\gamma -F_{f1}\sin\alpha -F_{f2}\sin\beta -F_{f3}\sin\gamma =0$$

(3) $$F_{ver}+N_{1}sin\alpha +N_{2}sin\beta +N_{3}\sin\gamma +F_{f1}\cos\alpha +F_{f2}\cos\beta +F_{f3}\cos\gamma =0$$

(4) $$M_{S}+F_{f1}R+F_{f2}R+F_{f3}R-M_{b}=0$$

where α,β,γ are the position angles of the first, second, and third guide pads;  R is the tool body radius.

### 2.3. Principle of Spindle Axis Deflection

**Figure 5:**
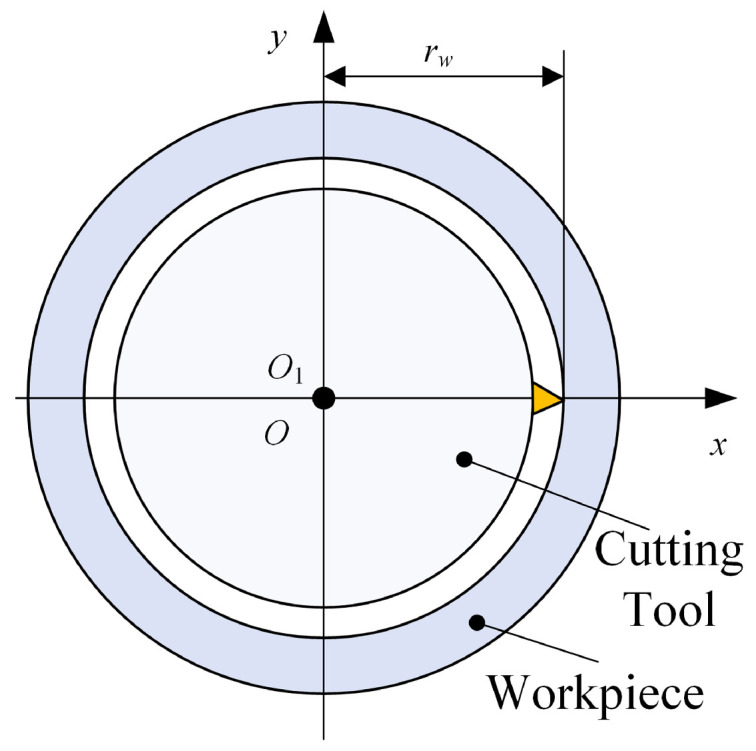
Processing process section.

Deep bottle hole machining adopts the method of workpiece rotation and tool feeding, as shown in Figure 5. Since the workpiece consistently rotates around the cross-sectional center O on the spindle, maintaining alignment with the axis of the deep blind hole-boring tool, this ensures the straightness of the machined deep blind hole.

In the machining method where the workpiece rotates and the tool feeds, the rotational speed of the deep hole internal profile cutting tool is 0, and the trajectory of its cutting edge can be expressed as

(5) $$\left\{\begin{array}{l} x=r_{w}\cos\omega_{w}t\\ y=r_{w}\sin\omega_{w}t\\ z=v_{f}t \end{array}\right.$$

where *x*, *y*, *z* are the coordinates of the tool tip in the Cartesian coordinate system; *t* is processing time; $\omega_{w}$ is the angular velocity of the workpiece; $r_{w}$ is the radius of the workpiece; $v_{f}$ is the tool cutting feed speed.

## 3. Tool Chip Evacuation Analysis

### 3.1. Chip Evacuation Mechanism

**Figure 6:**
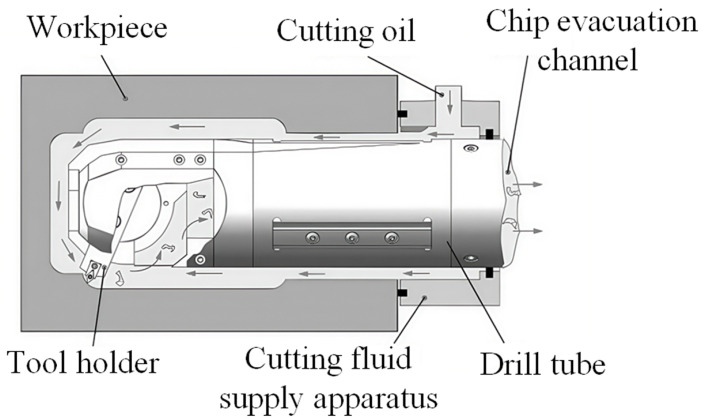
Schematic diagram of chip flow.

During deep blind hole boring, chips accumulate in the annular area between the tool and the workpiece. To prevent the chips from scratching the machined surface, the cutting fluid supply apparatus injects cutting fluid into the gap between the workpiece and the tool. The cutting fluid carries the chips out through the internal channel of the tool, as shown in Figure 6.

This chip evacuation process constitutes a typical solid–liquid two-phase flow dynamics problem. The chips, influenced by gravity and their own shape among other non-negligible factors, are non-uniformly distributed in the cutting fluid, forming the dispersed phase. The cutting fluid, acting as the continuous phase, fills the entire flow field, exhibiting continuity and incompressibility.

To accurately simulate the actual flow state of cutting fluid, the CFD simulation process typically defines boundary conditions as flow velocity. The Bernoulli equation effectively describes the relationship between cutting fluid velocity and pressure. Since cutting fluid can be simplified as an incompressible ideal fluid, the Bernoulli equation can be simplified as

(6) $$P+\frac{1}{2}\rho v^{2}+\rho gh=\mathrm{Constant}$$

where *P* is the static pressure of the cutting fluid; ρ is the density; ν is the velocity; g is the gravitational acceleration; h is the fluid domain height.

The deep bottle blind hole-boring process shares the same cutting fluid flow mechanism as typical deep hole machining processes like BTA drilling. Since the influence of fluid domain height variation is negligible, the cutting fluid velocity equation can be approximated as

(7) $$\nu =C\sqrt{\frac{P}{\rho }}$$

where *P* is the static pressure of cutting fluid; *C* is the empirical coefficient.

Actual viscous losses, non-ideal flow patterns, and machining interferences during deep bottle hole-boring operations all result in the “theoretical flow velocity” exceeding the “actual flow velocity”. In such cases, an empirical coefficient C (typically ranging between 0.6 and 0.9) must be introduced to ensure that the theoretically calculated coolant flow velocity aligns with real-world machining requirements. According to the above equation, the inlet velocity of the cutting fluid in the fluid domain boundary conditions should be selected within the range of 20~40 m/s.

### 3.2. Tool Chip Evacuation Channel Optimization

Cutting fluid plays the roles of cooling and chip evacuation in the deep blind hole-boring system. According to the requirements of pressure, flow velocity, etc., in the deep hole cutting environment, 69-1 emulsion is selected as the cutting fluid.

To investigate the mechanism of tool geometry’s influence on the flow field characteristics of cutting fluid under different flow rates, the cutting fluid inlet velocity was set at three gradients: 20, 30, and 40 m/s. Using the Fluent module in Ansys simulation software (18.0 version), the flow process of the cutting fluid was simulated to identify the critical locations where the fluid’s movement is most significantly impacted during its interaction with the tool. Subsequently, the tool structure was optimized to enhance the cutting fluid’s flow performance.

#### 3.2.1. Chip Evacuation Performance Analysis

The flow of cutting fluid during chip evacuation can be divided into three stages. In the first stage, within the annular flow-restricting section between the tool and the workpiece, the cutting fluid is easily obstructed by the guide pad and vibration-damping strips, leading to a reduction in flow velocity. During the second stage, as the cutting fluid enters the narrow area at the entrance of the chip evacuation channel, restricted space can trigger turbulent phenomena, causing significant fluctuations in fluid velocity. In the third stage, when the cutting fluid enters the latter half of the chip evacuation duct, the flow gradually transitions to laminar flow, resulting in a stabilized velocity.

**Figure 7:**
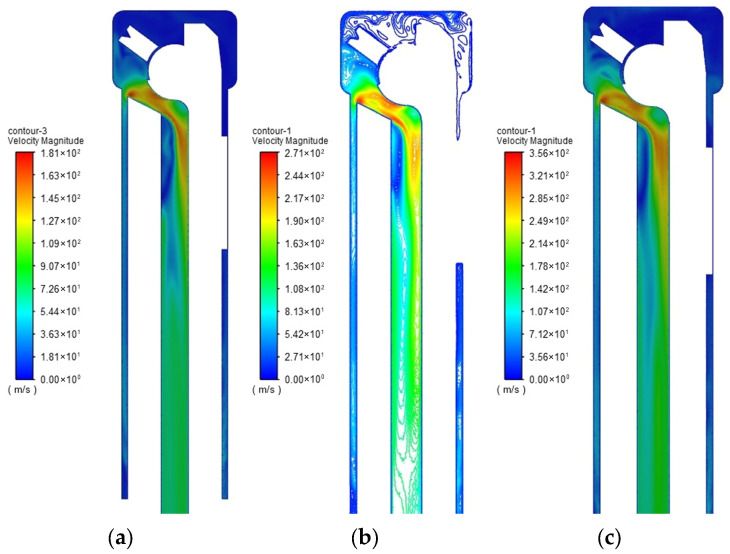
Cutting fluid flow rate diagram. (**a**) Flow velocity 20 m/s. (**b**) Flow velocity 30 m/s. (**c**) Flow velocity 40 m/s.

To more intuitively explore the changes in cutting fluid flow velocity within the fluid domain, the cutting fluid velocity contour of the vertical cross-section of the tool is selected, as shown in Figure 7.

From the observation of the cutting fluid velocity contour map, it can be seen that in the first stage, the overall turbulence intensity is low under different cutting fluid flow rates. After entering the second stage, when the inlet flow rate of the cutting fluid is 20 m/s, the turbulence intensity increases significantly at the turning of the chip evacuation channel inlet, mainly due to the poor transition design and the presence of sharp edges in this area. When the inlet flow rate is 30 m/s, part of the cutting fluid flows along the annular flow restriction section, bypasses the tool tip, and then flows to the chip evacuation channel inlet, forming vortices in the local area of the tool tip, which consumes the flow kinetic energy. When the inlet flow rate is 40 m/s, after converging at the chip evacuation channel inlet of the tool, it is blocked by the sharp edges of the inlet, forming a weak spiral flow. After entering the third stage, regardless of the change in flow rate, the cutting fluid gradually returns to a stable irrotational motion state after completely entering the chip evacuation channel.

The flow velocity of the cutting fluid directly affects the kinetic energy of chips during chip evacuation. A higher flow velocity can effectively enhance the drag force of cutting fluid on chips, promoting the rapid discharge of chips from the machining area. The cutting fluid flow velocities at each stage are shown in Table 1. The cutting fluid flows along the outer wall of the tool during the process of entering the chip evacuation channel, and the tool geometry directly affects the movement path of the cutting fluid. Especially at the inlet of the tool chip evacuation channel, the flow state of the cutting fluid is non-uniform with large velocity fluctuations, which can easily lead to chip accumulation.

**Figure 8:**
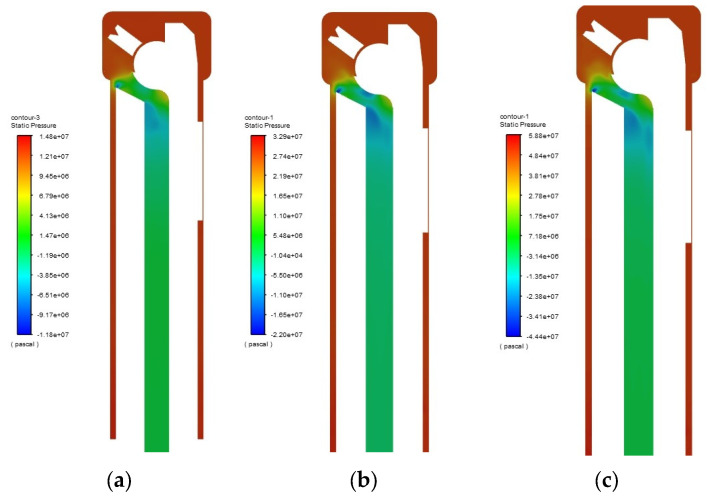
Pressure distribution inside the tool. (**a**) Flow velocity 20 m/s. (**b**) Flow velocity 30 m/s. (**c**) Flow velocity 40 m/s.

In this research on the real pressure of cutting fluid in a moving state, where the post-processing output here is static pressure, “±” only represents the level relative to the reference pressure. The pressure distribution of cutting fluid between the tool and the workpiece is shown in Figure 8.

In the figure, the red color represents the high-pressure cutting fluid area, which is mainly distributed between the tool and the workpiece; the blue color represents the low-pressure cutting fluid area, which is mainly distributed at the two corners of the chip evacuation inlet. The high-pressure cutting fluid can effectively penetrate the machining area, resulting in high chip evacuation efficiency, while the low-pressure cutting fluid area has weak chip-carrying capacity and low chip evacuation efficiency.

The cutting fluid pressure conditions at each stage are shown in Table 2. The movement of cutting fluid in the annular flow restriction section is relatively complex; a small amount of cutting fluid bypasses the top of the tool and enters the inlet, resulting in flow instability. At the inlet of the chip evacuation channel, the cutting fluid is obstructed by sharp corners, leading to severe pressure fluctuations and a significant decrease in chip evacuation power.

In summary, the sharp structures in both the external tool geometry and the internal chip evacuation channel entrance led to significant dissipation of the cutting fluid’s kinetic energy upon entering the channel. Therefore, based on the cutting fluid simulation results, further optimization of the tool’s structural parameters is required.

#### 3.2.2. Tool Structure Optimization

For the arc structure at the inlet of the chip evacuation channel, this paper adopts the variable-angle helix design method. Based on the simulation results, the chip evacuation inlet angle and inlet turning structure are adjusted to achieve chip evacuation optimization. To facilitate the optimization of the chip evacuation channel, as shown in the figure below, a polar coordinate system is established in the vertical cross-section of the tool, and the mathematical expression of the variable-angle helix is as follows:

(8) $$r=r_{1}e^{\theta \tan\alpha }$$

where r, $r_{1}$, $r_{2}$ is the radius of the spiral in polar coordinates and the entry/exit radii of the chip evacuation channel inlet; e is the base of the natural logarithm; α is the angular constant; θ,φ, is the polar angle in the polar coordinate system and the entrance wrap angle.

The boundary condition is $\left\{\begin{array}{l} \theta =0,r=r_{1}\\ \theta =\varphi ,r=r_{2} \end{array}\right\}$

Establish the equation for the red contour of the tool:

(9) $$r^{2}+2\left[R_{1}\cos(\theta +\beta )-r_{1}\cos\theta \right]\cdot r+r_{1}(r_{1}-2R_{1}\cos\beta )=0$$

where β is helix setting angle; $R_{1}$ is the reference radius.

Substitute the polar angle expression $\alpha =\alpha \left(\theta \right)$ into Equation (9) above to obtain

(10) $$tg\beta =tg\alpha +\theta (1+tg^{2}\alpha )\frac{d\alpha }{d\theta }$$

According to Equation (10) above, the variation law between the polar angle *α* of the arc helix and the helix placement angle *β* can be derived:

(11) $$\beta =tg^{-1}(\alpha \theta^{k}+b)$$

And by substituting the boundary conditions $\left\{\begin{array}{c} \theta =0,\beta =\beta_{1}\\ \theta =\varphi ,\beta =\beta_{2} \end{array}\right.$ into Equation (11) above, the formula for the placement angle *β* can be obtained:

(12) $$\beta =tg^{-1}(\frac{tg\beta_{2}-tg\beta_{1}}{\varphi^{k}}\theta^{k}+tg\beta_{1})$$

where $\beta_{1}$ and $\beta_{2}$ are the inlet and outlet pitch angles of the spiral line, respectively.

The expression for k in Equation (12) is as follows:

(13) $$k=\frac{(tg\beta_{2}-tg\beta_{1})\varphi }{\ln(r_{2}/r_{1})-\varphi tg\beta_{1}}-1$$

And by differentiating the above equation, the formula for the radius of curvature of the helix is obtained:

(14) $$R=\frac{{\left(r^{2}+r^{\prime 2}\right)}^{3/2}}{r^{2}+2r^{\prime 2}-rr^{\prime \prime }}=\frac{A{\left(1+B^{2}\right)}^{3/2}}{1+B^{2}-C}$$

The chip evacuation entrance arc derived from the helical angle *β* and curvature radius formula is optimized by replacing the sharp structure at the entrance turn of the chip evacuation channel with a smooth arc curve. This arc design not only effectively reduces kinetic energy loss when cutting fluid carries chips into the chip evacuation channel but also significantly weakens fluid pressure fluctuations, thereby accelerating cutting fluid flow velocity and enhancing the overall chip evacuation efficiency of the tool.

Analysis of cutting fluid velocity contour maps reveals that when cutting fluid flows over the top of the tool, part of the fluid generates vortices in the gap between the tool top and the workpiece, leading to kinetic energy attenuation during fluid flow. To address this, the tool top structure is optimized by inwardly reducing the upper cover by 5 mm to expand the cutting fluid flow space, thereby improving fluid circulation performance and boosting chip evacuation efficiency.

**Figure 9:**
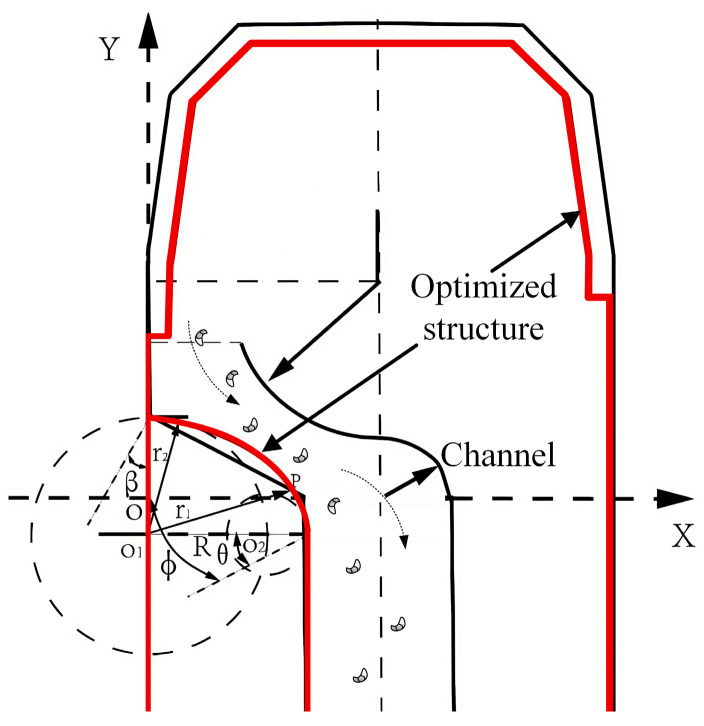
Tool structure comparison.

The structural improvements to the tool are illustrated in Figure 9, where the black outline represents the original tool contour and the red lines indicate the optimized structure.

### 3.3. Chip Evacuation Simulation

#### 3.3.1. CFD-DEM Coupled Calculation

In CFD-DEM coupled calculations, the governing equations of the continuous phase of the cutting fluid and the discrete phase of chips, as well as the two-way data exchange mechanism, are as follows:

(1) Governing equations of the continuous phase

The cutting fluid, as the continuous phase, primarily serves to transport chips and provide cooling and lubrication. The Navier–Stokes equations can accurately describe the fundamental motion of fluids. The flow of cutting fluid must adhere to the mass conservation law and momentum conservation law:

(15) $$\frac{\partial (\alpha_{f}\rho_{f})}{\partial t}+\nabla \cdot (\alpha_{f}\rho_{f}u_{f})=0$$

(16) $$\frac{\partial (\alpha_{f}\rho_{f}u_{f})}{\partial t}+\nabla \cdot (\alpha_{f}\rho_{f}u_{f}u_{f})=-\alpha_{f}\nabla p+\nabla \cdot \tau_{f}+\alpha_{f}\rho_{f}g+S_{p}$$

where $\alpha_{f}$ is the volume fraction of the cutting fluid in the two-phase flow; $\rho_{f}$ is the cutting fluid density; $u_{f}$ is the cutting fluid velocity vector; $S_{p}$ is the momentum exchange between continuous and discrete phases; *g* is gravitational acceleration; $\tau_{f}$ is the viscous stress tensor.

(2) Discrete phase control equation

The force distribution acting on chips during cutting fluid flow is relatively complex. To accurately characterize the trajectory of chips in solid–liquid two-phase flow, momentum and angular momentum conservation equations for particles are established to solve for particle velocity and displacement. In solid–liquid two-phase flow systems, the momentum and angular momentum conservation equations corresponding to the collision process between chip particles and cutting fluid particles are formulated as follows:

(17) $$m_{i}\frac{d\nu_{i}}{dt}=F_{\mathrm{fluid}}+F_{\mathrm{contact}}+F_{\mathrm{volume}}=\sum_{j}(F_{n,ij}+F_{t,ij})+m_{i}g+F_{fp,i}$$

(18) $$\frac{d}{dt}I_{i}\omega_{i}=\sum_{j}(r_{i}\times F_{t,ij}+M_{i})$$

where $m_{i}$ and $\nu_{i}$ are the chip mass and velocity; F is the force exerted by the object of study on other objects; $F_{n,ij}$ is the normal contact force between two particles; $F_{t,ij}$ is the tangential contact force between two particles; *I_i_* and $\omega_{i}$ are the moment of inertia and angular velocity of the chip; $F_{fp,i}$ is the force exerted by the cutting fluid on the chip; $r_{i}$ is position vector; $M_{i}$ is the rolling friction torque between two particles.

If chips move in a uniform cutting fluid flow field, only considering the effect of resistance force and gravity on the chips, and the chip resistance obeys Stokes’ law, the chip momentum equation can be derived:

(19) $$\frac{1}{6}\pi d_{p}^{3}\rho_{p}\frac{du_{pi}}{dt}=3\pi \mu_{f}d_{p}\left(u_{fi}-u_{pi}\right)+\frac{1}{6}\pi d_{p}^{3}\rho_{p}g_{i}$$

where dp is the particle diameter; $\rho_{p}$ is the particle density; $u_{pi}$ is the velocity of particles in the *i* direction; t is the time; $\mu_{f}$ is the dynamic viscosity of the fluid; $u_{fi}$ is the velocity of the fluid in the *i* direction; $\rho_{f}$ is the density of the fluid; $g_{i}$ is the gravitational acceleration component in the *i* direction.

From this we can get

(20) $$\begin{array}{cc} \frac{du_{pi}}{dt} & =\frac{1}{\tau_{rp}}\left(u_{fi}-u_{pi}\right)+g_{i}\\ & \Rightarrow d\left(u_{pi}e^{\frac{t}{\tau_{m}}}\right)=\left(\frac{u_{fi}}{\tau_{rp}}+g_{i}\right)e^{\frac{t}{\tau_{mp}}}dt\\ & \Rightarrow u_{pi}=\left(u_{fi}+\tau_{rp}g_{i}\right)+C_{1}e^{\frac{t}{\tau_{p}}} \end{array}$$

where $\tau_{rp}$ is the relaxation time; $\tau_{mp}$ is the momentum transfer time constant; $\tau_{m}$ is the relaxation time; $\tau_{p}$ is the characteristic time of particles; $C_{1}$ is the integration constant.

When *t* = 0, $u_{pi}=u_{pi0}$, it can be obtained that

(21) $$C_{1}=u_{pi0}-u_{fi}-\tau_{rp}g_{i}$$

The expression for the chip velocity can be obtained:

(22) $$u_{pi}=u_{fi}+\tau_{rp}g_{i}+\left(u_{pi0}-u_{fi}-\tau_{rp}g_{i}\right)e^{\frac{-t}{\tau_{rp}}}$$

When t→∞, $u_{pi}\to u_{fi}+\tau_{rp}g_{i}=\mathrm{const}$, at this point, the chip acceleration can be obtained $\frac{du_{pi}}{dt}=0$. From the above expression for the chip velocity, it can be converted into the equation of motion for the chip trajectory:

(23) $$\frac{dx_{pi}}{dt}=u_{fi}+\tau_{rp}g_{i}+\left(u_{pi0}-u_{fi}-\tau_{rp}g_{i}\right)e^{\frac{-t}{\tau_{rp}}}$$

where $x_{pi}$ is the chip movement trajectory coordinate.

By integrating the above equation of motion for the chip trajectory, the chip trajectory equation can be obtained:

(24) $$x_{pi}=\left(u_{fi}+\tau_{rp}g_{i}\right)t-\tau_{rp}\left(u_{pi0}-u_{fi}-\tau_{rp}g_{i}\right)e^{\frac{-t}{\tau_{rp}}}+C_{2}$$

When t=0, $x_{pi}=x_{pi0}$ can be obtained:

(25) $$C_{2}=x_{pi0}+\tau_{rp}\left(u_{pi0}-u_{fi}-\tau_{rp}g_{i}\right)$$

Finally, the chip trajectory equation can be obtained:

(26) $$x_{pi}=x_{pi0}+\left(u_{fi}+\tau_{p}g_{i}\right)t+\tau_{p}\left(u_{pi0}-u_{fi}-\tau_{rp}g_{i}\right)\left(1-e^{\frac{-t}{\tau_{pp}}}\right)$$

where $x_{pi0}$ is the initial position of the particle in the *i* direction; $u_{pi0}$ is the initial velocity of particles in the *i* direction; $\tau_{pp}$ is the particle-particle interaction time constant.

Chips, as the discrete phase, interpenetrate with the cutting fluid as the continuous phase. Thus, the discrete phase satisfies the pseudo-fluid concept. Chip particles are subjected to the interaction of various forces from the cutting fluid, mainly including drag force, pressure gradient force, Basset force, and lift force.

(1) Drag force is mainly the momentum exchange between the solid–liquid two-phase flow, and its calculation method is as follows:

(27) $$F_{d}=\frac{1}{2}C_{d}\rho A_{p}\left|\nu_{s}\right|\nu_{s}$$

where $F_{d}$ is the drag Force; $A_{p}$ is the projected area of the chip; $v_{s}$ is the slip speed of chips; $C_{d}$ is the drag coefficient between the solid–liquid two-phase flow.

(2) The pressure gradient force is an additional force on a single chip particle caused by the pressure gradient in the cutting fluid flow field, and its calculation method is as follows:

(28) $$F_{P}=-\frac{4}{3}\pi r_{p}^{3}\nabla p_{f}$$

where $F_{P}$ is the pressure gradient force; $r_{p}$ is the radius of particles; $\nabla p_{f}$ is the pressure gradient of fluid.

(3) Basset force is an unsteady resistance force caused by fluid viscosity, related to the time integral of the historical acceleration of particle motion when chip particles move unsteadily in viscous fluid, and its calculation method is as follows:

(29) $$F_{B}=K_{B}r_{p}^{2}\sqrt{\pi \rho_{f}\mu_{f}}\int_{0}^{t}\frac{1}{\sqrt{t-\tau }}\frac{d}{dt}\left(u_{f}-u_{p}\right)d\tau$$

where $F_{B}$ is the basset force; $K_{B}$ is the basset force coefficient; τ is the time variable.

In the equation, $K_{B}=6$

(4) The lift force originates from the lateral force exerted on the chips by the shear flow field of the cutting fluid, and its calculation method is as follows:

(30) $$F_{LR}=\frac{\rho \pi }{8}d_{p}^{2}C_{LR}|u_{slip}|\frac{\Omega \times u_{slip}}{\left|\Omega \right|}$$

where $F_{LR}$ is the lift force; $C_{LR}$ is the lift coefficient; Ω is the vector of the angular velocity of the vorticity field; $u_{slip}$ is the relative velocity vector between the particle and the fluid.

The interaction forces between chips and cutting fluid directly influence the motion trajectory, velocity, and direction of chips. Accurately simulating chip motion in fluid environments through CFD-DEM coupled computation facilitates the prediction of chip trajectories and reveals their accumulation patterns within the tool interior.

#### 3.3.2. Chip Movement Simulation

Compared with the traditional chip evacuation simulation that solely models the flow of cutting fluid, this paper employs CFD-DEM coupling simulation. EDEM (Discrete Element Method) software (2018 version) is used to construct the properties and contact models of chip particles, and the DEM solver updates the positions, accelerations, and velocities of particles in the dispersed phase. Meanwhile, Fluent (18.0 version) invokes UDF (User-Defined Function) to compute the transient flow field and generate particle distributions. This approach breaks through the limitations of one-way interaction between the continuous and discrete phases, achieving high-precision modeling of the multi-physical field interaction between cutting fluid and chips, and accurately simulating the motion of chips in the cutting fluid.

**Figure 10:**
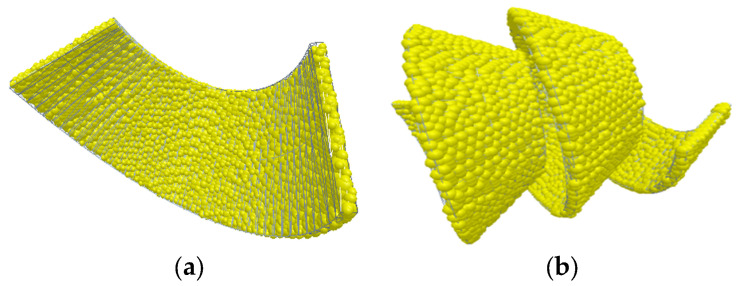
Chip model. (**a**) C-type chip. (**b**) Helical curly chip.

The specific simulation process is as follows:

(1) Chip particle generation: Using the particle scanning function in EDEM, single-sphere model particles with a diameter of 0.1 mm are adopted to establish C-type chip and spiral chip models, with the smoothness set to 1. The generated chip models are shown in Figure 10.

Add a particle generation factory, refer to the chip generation speed, set the particle injector type to static, and set the injection speed to 1 m/s in the *x*-axis, −1 m/s in the *y*-axis, and 0.5 m/s in the *z*-axis; the number of generated particles is 10, and the chip particle parameters are defined as shown in Table 3.

After modeling the chip particles, set the particle factory to physical state, output the generated chip results as an input.dem file, and set the simulation time to 0 s. Change the injector to virtual state, which can collide with geometric surfaces, so that the particles can move within the entire computational domain.

(2) Coupling environment configuration: Install EDEM-Fluent Coupling Interface and configure environment variables: EDEM_COUPLING_INC and EDEM_COUPLING_LIB.
(3) Fluid model establishment: Add the particle source term via UDF, synchronize the chip parameters set in EDEM, and add physical models of Saffman lift force and pressure gradient force. The turbulence model adopts the SST k-ω model (Shear Stress Transport k-ω model). EDEM can only simulate transient states, so Fluent is also used.
(4) Coupling interface configuration: The EDEM time step is set to 2 × 10^−7^ s, so that the fixed time step ratio is less than 20%. The Fluent time step is an integer multiple of the EDEM time step.
(5) CFD solving of fluid field: Solve the modified Navier–Stokes equations (including porosity and momentum source terms) and update the flow field data.
(6) Data transfer: Interpolate the cutting fluid velocity and pressure to the positions of chip particles, and calculate the fluid force acting on the chips.
(7) Check fluid residuals: The continuity residual < 10^−4^, the momentum residual < 10^−4^, and the fluctuation of particle kinetic energy < 5%, so as to determine whether to enter the next time step.

### 3.4. Effect of Chip Shape

This study is based on the multi-condition flow field comparative analysis method, setting the inlet flow velocity of cutting fluid to three characteristic gradients: 20, 30, and 40 m/s. Through the EDEM-Fluent two-way coupling simulation platform, the migration trajectories of spiral chips and C-type chips in the tool flow channel are captured in real time.

**Figure 11:**
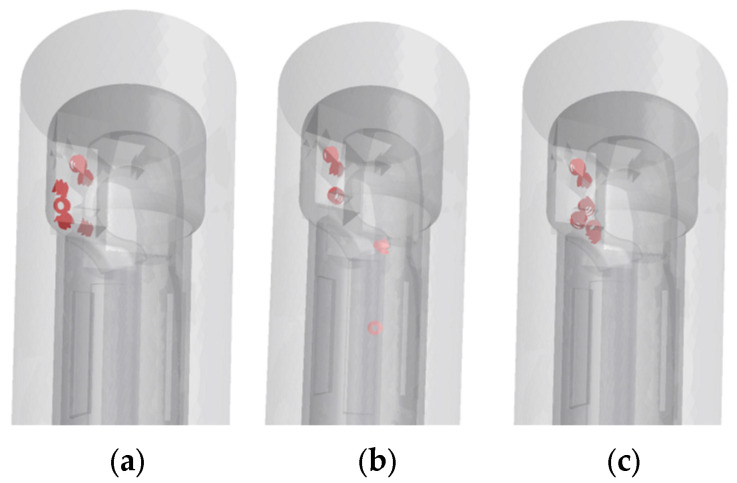
Spiral chip movement conditions: (**a**) 20 m/s; (**b**) 30 m/s; (**c**) 40 m/s.

At the initial flow velocity of 20 m/s (Figure 11a), although the basic chip evacuation function can be maintained, due to insufficient flow velocity, some chips are temporarily retained near the tool tip. When the flow velocity is increased to 30 m/s (Figure 11b), the spiral chips are fully unfolded under the action of high-speed shear, the flow uniformity in the chip evacuation channel is improved, and the evacuation efficiency reaches the optimal state. When the flow velocity is increased to 40 m/s (Figure 11c), the fluid enters the strongly turbulent region, and a flow dead zone is formed in the geometric mutation region between the tool shank and the turntable due to local pressure gradient imbalance, resulting in chip accumulation.

**Figure 12:**
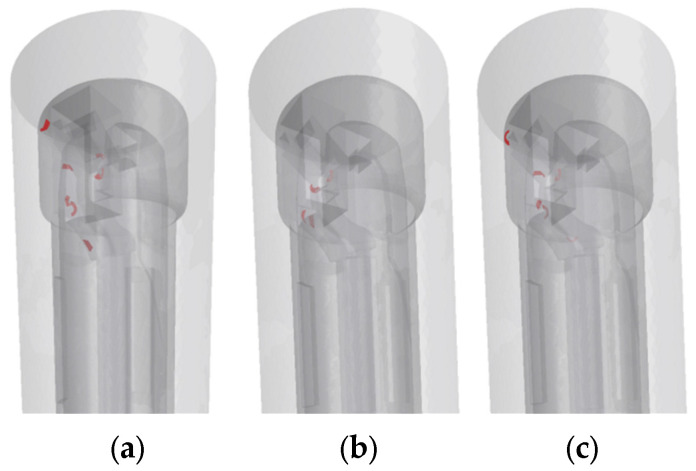
C-type chip movement condition: (**a**) 20 m/s; (**b**) 30 m/s; (**c**) 40 m/s.

Under the low-speed condition of 20 m/s (Figure 12a), the fluid–solid coupling effect has not yet significantly dominated the particle motion, but the chips still show local disordered deviation due to insufficient inertia, initially forming a dispersion trend. When the speed increases to 30 m/s (Figure 12b), the enhanced turbulent kinetic energy leads to intensified flow field disturbance, and the particle phase deviates from the stable flow lines under the high-speed shear effect. The fluid–solid coupling effect further expands the dispersion range of the chips. Under the high-speed working condition of 40 m/s (Figure 12c), the cutting fluid turbulence enters a strongly nonlinear region, the frequency of vortex structures and particle collisions increases significantly, and finally, a global non-directional dispersion phenomenon is formed in the chip evacuation channel.

In summary, under the same environment, the chip evacuation effect of helical curled chips is significantly better than that of C-type chips. Its continuous helical structure generates a centrifugal force effect, enabling the chips to be discharged directionally along the predetermined trajectory and effectively avoiding the common accumulation phenomenon of C-type chips. The removal speed of helical chips is increased by approximately 40% compared with that of C-type chips. 

## 4. Processing Test

### 4.1. Test Scheme

**Figure 13:**
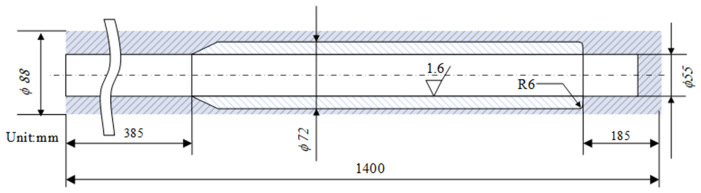
Finished tube.

The material of the test workpiece is a 07Cr16Ni6 low-pressure turbine shaft of a certain aero-engine. The tube’s outer dimensions are ϕ 88 mm × 1400 mm and its inner hole dimension is ϕ55 mm. It is necessary to use the boring tool to process this deep hole tube. The specific machining requirements are as follows: expand the inner hole size to ϕ72 mm while keeping its outer dimensions unchanged, as shown in Figure 13. The final deep blind hole wall roughness is required to reach Ra 1.6 µm.

**Figure 14:**
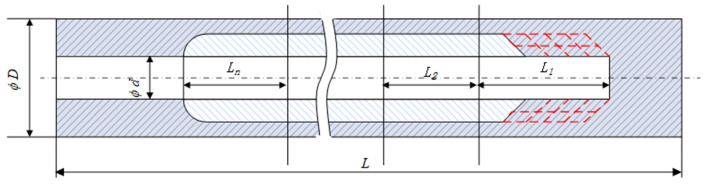
Schematic diagram of segmented cutting.

**Figure 15:**
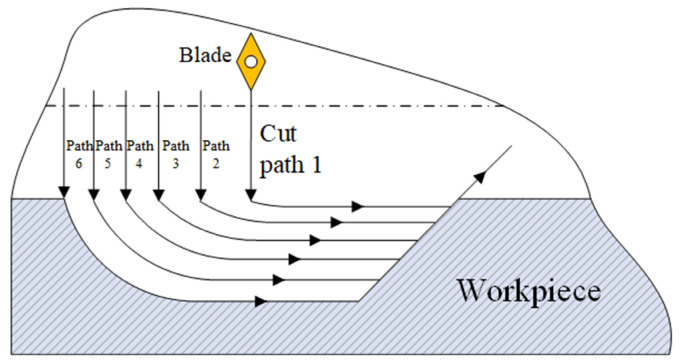
Schematic diagram of layered cutting.

Due to the complex structure of the deep blind hole bottle cavity, an excessive cut depth during machining will cause the cutter body to lack support and guidance. Meanwhile, excessive cutting force easily leads to tool vibration, which reduces the accuracy of the inner surface of the hole. Therefore, a segmented and layered boring process is adopted, as shown in Figure 14. The entire deep blind hole-boring process is divided into multiple segments. The tool enters the deep hole through the pilot hole and sequentially performs segmented and layered machining on L_1_, L_2_, …, L*n* from right to left. To avoid seam marks between adjacent segments, the tool path of each segment overlaps with that of the previous segment, as shown in Figure 15.

Based on the aforementioned simulation results, to further investigate chip evacuation control and machining accuracy in actual production processes, and to validate the rationality of segmented and layered boring technology, machining experiments were conducted. According to practical machining experience, the depth of cut ap was set to 0.2 mm, while the cutting speed *v*, feed rate *f*, and coolant flow rate *Q* were selected as variables. The study examined chip morphology and chip evacuation effectiveness under different combinations of cutting parameters. The specific process parameters selected are shown in Table 4.

The cutting fluid selected is the water-based coolant (69-1 emulsion) used in the simulation test. Selection of inserts is directly related to chip morphology. The insert material selected in this test is YG8 cemented carbide, and its specific parameters are shown in Table 5. The above choices are based on Sandvik’s deep hole drilling manual.

### 4.2. Experimental Conditions

**Figure 16:**
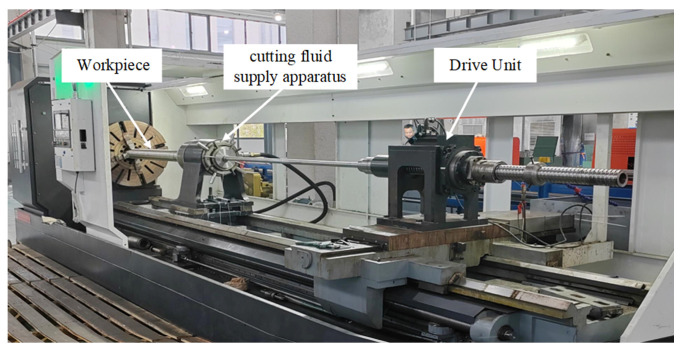
SKZY-DH-5 smart control deep hole machine tool.

The deep blind hole-boring tool was installed on the SKZY-DH-5 intelligent control deep hole-boring machine for testing. The length of its drill rod is 5000 mm, which can complete deep hole machining with a large depth-to-diameter ratio, as shown in Figure 16.

### 4.3. Experimental Results

#### 4.3.1. Analysis of Chip Evacuation Situation

The chip shape and chip evacuation from the test machining will be analyzed, to provide a reference basis for subsequent process parameter settings. The results of chip morphology and chip evacuation conditions under different process parameters in each group of experiments are shown in Table 6.

**Figure 17:**
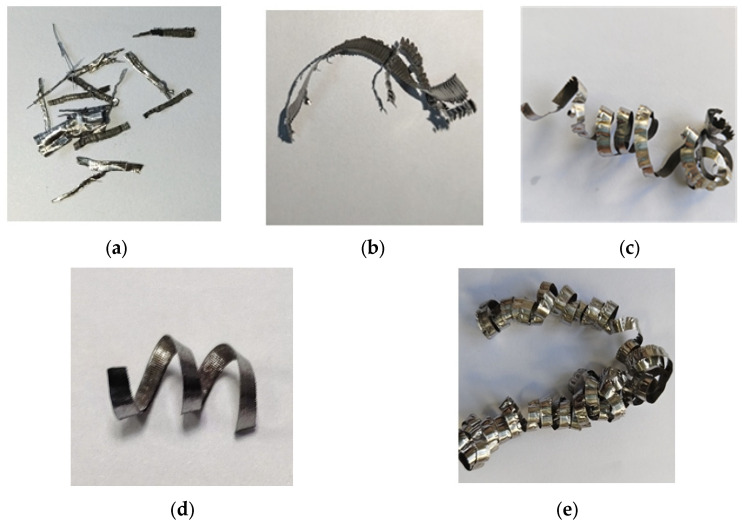
Experimental chip morphology: (**a**) acicular chips; (**b**) folded curly chips; (**c**) long folded curly chips; (**d**) helical curly chips; (**e**) long folded curly chips.

The specific chip morphologies are shown in Figure 17.

The centrifugal force of the helical curly chips is concentrated and directionally stable, providing the chips with a “self-guiding” ability and reducing the probability of random collisions with the channel walls. They also have the minimum curling radius, and their continuous helical structure can undergo “flexible deformation”. When passing through channel corners, they can adaptively adjust their posture to avoid getting stuck due to size constraints. Additionally, the continuous helical surface of the helical curly chips forms surface contact with the cutting fluid, allowing the cutting fluid to evenly act on the chip surface and avoiding stagnation caused by insufficient local force.

To sum up, Group 4 is selected as the experimental machining parameters. A set of optimal cutting parameters for deep blind hole boring is (*v*, *f*, *Q*) = (22 m/min, 0.018 mm/r, 180 L/min), with the chip morphology being helical curled chips with the highest chip evacuation efficiency.

#### 4.3.2. Surface Quality Analysis

**Figure 18:**
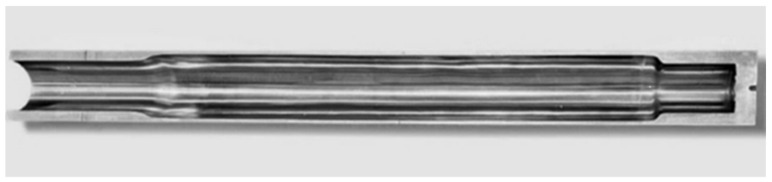
Section view of finished trial sample.

Based on the obtained machining parameters, the trial-produced finished workpiece is shown in Figure 18. The inner surface roughness of the workpiece was measured using the Mitutoyo SJ-220 surface roughness tester (Mitutoyo, Kawasaki, Japan). The surface roughness Ra at the entrance was 0.6 µm, meeting the processing requirement of Ra ≤ 0.8 µm. The surface roughness Ra of the intermediate tapered hole was 0.9 µm, satisfying the processing requirement of Ra ≤ 1.2 µm. The overall results met the processing standards. The surface roughness Ra of the transition arcs at both ends reached 1.6 µm. The slightly higher roughness here was due to the tool requiring continuous adjustment of radial feed during machining, leading to fluctuating cutting forces and increased risk of vibration, which degraded surface quality.

#### 4.3.3. Axis Line Deflection Analysis

**Figure 19:**
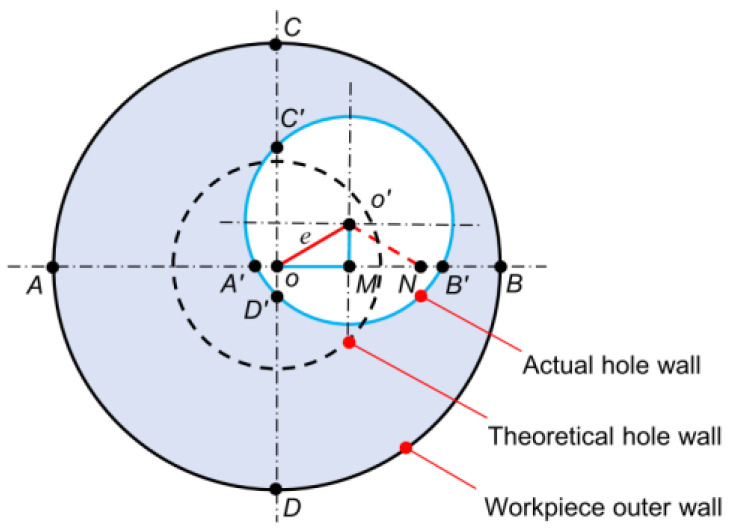
Principle of hole axis line deflection.

The boring process of deep bottle holes belongs to the category of deep hole boring and can correct the axis alignment of the hole [22]. The principle of axis deviation is shown in Figure 19.

The geometric model of eccentricity is as follows:

(31) $$e=OO^{\prime }=\sqrt{OM^{2}+O^{\prime }M^{2}}=\sqrt{{\left(\frac{AA^{\prime }-BB^{\prime }}{2}\right)}^{2}+{\left(\frac{DD^{\prime }-CC^{\prime }}{2}\right)}^{2}}$$

To verify the deviation correction effect of the deep blind hole-boring process, the eccentricities e and $e_{1}$ of the deep hole tube before and after machining were measured. The specific measurement steps were as follows: Starting from 200 mm from the right end face of the tube, at intervals of 100 mm, the JK-5C ultrasonic thickness gauge (Jiekesi, Shenzhen, China) was used to measure the wall thickness at four points on the circumference of the workpiece. Thus, the eccentricity of different cross-sections of the machined hole was calculated using Equation (31). To ensure the reliability of the wall thickness values, the wall thickness at each point was measured three times, and the average value was taken. The wall thickness before machining is denoted by $AA^{\prime }$, $BB^{\prime }$, ${CC}^{\prime }$, ${DD}^{\prime }$, and the eccentricity is denoted by *e*; the wall thickness after machining is denoted by $A_{1}A_{1}^{\prime }$, $B_{1}B_{1}^{\prime }$, ${C_{1}C}_{1}^{\prime }$, ${D_{1}D}_{1}^{\prime }$, and the eccentricity is denoted by *e*_1_. Finally, the specific measurement and calculation results calculated by Equation (31) are shown in Table 7.

The hole axis deviation curve shows an increasing trend. This is because the hole depth increases after deep hole drilling. As the hole depth increases, the drill rod will gradually elongate, and its rigidity is bound to gradually decrease. At this time, the mechanical vibration of the boring bar will also gradually increase, and the degree of hole axis deviation will increase accordingly.

It can be found that the maximum eccentricity before machining reaches 0.57 mm, and after deep blind hole boring, the maximum eccentricity is 0.26 mm. The hole axis deviation is significantly reduced, which verifies the deviation correction effect of this type of machining method and meets the straightness error requirement.

## 5. Conclusions

(1) This study developed a dedicated boring tool specifically for deep blind hole-boring operations and designed a workpiece–tool–machine integrated processing system based on this tool, enabling a multi-axis collaborative machining mode to ultimately achieve the deep blind hole boring of deep bottle holes.
(2) Through simulation of cutting fluid flow within the tool, the chip evacuation channel design was optimized to significantly improve fluid flow efficiency. CFD-DEM coupled calculations were employed for solid–liquid two-phase flow simulations to model the chip evacuation processes of C-shaped and helical chips, revealing that helical chips exhibited superior evacuation performance under all tested flow velocity conditions compared to C-shaped chips.
(3) Targeting the structural characteristics of deep bottle holes, a segmented and layered deep hole-boring process was proposed. Using an aircraft engine low-pressure turbine shaft as the test object, experimental validation demonstrated that the deep blind hole-boring tool exhibited excellent chip evacuation performance, with an internal surface roughness of 0.9 µm meeting design requirements. The workpiece coaxiality eccentricity was reduced by 54.39% compared to the raw material state, validating the feasibility and effectiveness of the tool system and machining solution.

## Figures and Tables

**Table 1:** Flow velocity situation of cutting fluid at each stage.

Different Regions	Flow Velocity		
	20 m/s	30 m/s	40 m/s
Upper flow velocity (m/s)	0~18.2	0~27.1	0~35.6
The channel entrance (m/s)	181~109	136~271	178~285
Outlet flow velocity (m/s)	72.6	108	142

**Table 2:** Pressure situation of cutting fluid at each stage.

Different Regions	Flow Velocity		
	20 m/s	30 m/s	40 m/s
The annular region (MPa)	12.1	27.4	48.4
The entrance of the chip evacuation channel (MPa)	−6.51~6.79	−22~11	−44.4~7.18
Outlet pressure (MPa)	−1.19	−0.0104	−3.14

**Table 3:** Chip particle parameter.

Parameter	Particle Density (kg/m^3^)	Particle Diameter (mm)	Young’s Modulus (MPa)	Poisson’s Ratio	Coefficient of Restitution	Coefficient of Static Friction
Numerical value	7720	0.1	1	0.28	0.1	0.8

**Table 4:** Test factors and levels.

Test Group Serial Number	Cutting Speed *v* (m/min)	Feed Rate *f* (mm/r)	Cutting Fluid Flow Velocity *Q* (L/min)
1	15.5	0.01	180
2	19.6	0.01	125
3	19.6	0.026	180
4	22.0	0.018	180
5	23.7	0.026	125

**Table 5:** Insert parameters.

Insert	Model	Material	$\gamma_{0}$	$\alpha_{0}$	$\lambda_{s}$	$\kappa_{r}$	$R_{n}$
Parameter	CCMT060204	YG8	7°	10°	0°	55°	0.4 mm

**Table 6:** Experimental chip situation.

Experimental Groups	Chip Morphologies	$\mathrm{Curl}\;\mathrm{Radius}\;r_{f}$ (mm)	Chip Evacuation Situation
1	acicular chips	4.71	unable to evacuate chips normally
2	folded curly chips	5.42	frequent chip blocking
3	long folded curly chips	5.25	slight chip blocking
4	helical curly chips	3.9	high chip evacuation efficiency
5	long folded curly chips	5.45	slight chip blocking

**Table 7:** Wall thickness measurement data.

Drilling Depth (mm)	Wall Thickness (mm)								Eccentricity e (mm)	$\mathrm{Eccentricity}\;e_{1}$ (mm)
	$AA^{\prime }$	$BB^{\prime }$	${CC}^{\prime }$	${DD}^{\prime }$	$A_{1}A_{1}^{\prime }$	$B_{1}B_{1}^{\prime }$	${C_{1}C}_{1}^{\prime }$	${D_{1}D}_{1}^{\prime }$		
400	16.9	16.21	16.31	16.63	8.28	7.96	8.13	7.85	0.38	0.21
600	16.88	16.16	16.38	16.7	8.25	7.93	8.16	7.88	0.39	0.21
800	16.94	16.14	16.43	16.72	8.27	7.88	8.14	7.9	0.43	0.23
1000	17.04	16.08	16.52	16.67	8.27	7.85	8.12	7.89	0.49	0.24
1200	17.11	15.98	16.57	16.77	8.26	7.83	8.16	7.95	0.57	0.24

## Data Availability

The original contributions presented in this study are included in the article. Further inquiries can be directed to the corresponding author.
